# Artificial intelligence to support debriefing in simulation-based healthcare education: a scoping review

**DOI:** 10.1186/s41077-026-00447-6

**Published:** 2026-06-25

**Authors:** Aseelah Alnazawi, Mohammed Almarhabi

**Affiliations:** 1 Jeddah Clinical Skills and Simulation Center, College of Medicine, King Saud Bin Abdulaziz University for Health Sciences (KSAU-HS), King Abdulaziz Medical City, Jeddah, Saudi Arabia; 2 Florence Nightingale Faculty of Nursing, Midwifery & Palliative Care, King’s College London, 57 Waterloo Road, London, SE1 8WA United Kingdom; 3 Emergency Medical Services, College of Applied Medical Sciences, Umm Al-Qura University, Makkah, Saudi Arabia

**Keywords:** Artificial intelligence, Debriefing, Simulation-based education, Healthcare simulation, Health professions education, Scoping review

## Abstract

**Background:**

Artificial intelligence is increasingly being integrated into healthcare education and simulation-based education. However, its role in supporting the debriefing phase of simulation remains underexplored and inconsistently described. This scoping review aimed to map the existing literature on the use of artificial intelligence to support debriefing in simulation-based healthcare education.

**Methods:**

A scoping review was conducted in accordance with Arksey and O’Malley’s framework and Joanna Briggs Institute guidance and reported in line with PRISMA-ScR. MEDLINE, Scopus, Web of Science, and CINAHL were searched without date restrictions. Eligible studies examined the use of artificial intelligence to support debriefing-related processes within healthcare simulation. Data were charted using a structured extraction form and synthesised descriptively and thematically.

**Results:**

Seven studies published between 2023 and 2026 met the inclusion criteria. Studies were conducted in the United States, Switzerland, Chile, and South Korea. Artificial intelligence applications clustered into three domains: communication and performance analytics using speech recognition and natural language processing; generative artificial intelligence systems supporting facilitator feedback and structured report generation; and learner-facing reflective dialogue systems. Across the included studies, artificial intelligence was mainly positioned as an adjunct to human facilitation rather than as a replacement for facilitators. Reported outcomes focused primarily on feasibility, usability, technical accuracy, and perceived educational value, with limited evidence of objective improvements in learner performance or clinical outcomes.

**Conclusions:**

Artificial intelligence is emerging as a supportive tool for debriefing in simulation-based healthcare education. Current evidence remains limited, exploratory, and largely single-institutional, indicating the need for more rigorous research on educational effectiveness, ethical implementation, and the continuing role of human facilitation.

**Supplementary Information:**

The online version contains supplementary material available at https://doi.org/10.1186/s41077-026-00447-6.

## Introduction

Artificial intelligence (AI) broadly refers to computational systems designed to perform tasks that typically require human intelligence, such as pattern recognition, decision-making, and natural language processing, often through algorithms that learn from large datasets [1, 2]. Advances in machine learning, natural language processing, generative AI, and AI-driven analytics have accelerated the integration of AI technologies across healthcare systems and educational environments [1, 3–5]. Within healthcare and health professions education, AI applications increasingly include clinical decision support, educational content generation, automated assessment, personalised learning support, and feedback generation [3, 5]. Simulation-based education is an established pedagogical approach for developing clinical competence in a safe and controlled learning environment [6, 7]. Simulation allows learners and teams to practise clinical reasoning, technical skills, and teamwork behaviours without risk to patients, while enabling educators to observe performance and support learning through structured feedback and reflection [8–10]. As educational technologies evolve, there is increasing interest in how AI-enabled tools may be integrated into simulation-based education to support processes such as performance analysis, feedback generation, and learner reflection [11–13].

Debriefing is a central component of simulation-based education and is widely considered the primary process through which learning is consolidated following simulation activities [6, 7]. In this review, debriefing is understood as a structured, facilitated reflective process that enables learners to analyse their actions, clinical reasoning, and team performance in relation to learning objectives. Debriefing should not be reduced to feedback delivery alone; rather, it supports guided reflection, meaning-making, and the translation of simulation experiences into future practice [6, 7, 9]. Therefore, tools that support performance review, feedback, reflective dialogue, and facilitator preparation may have important implications for the quality and consistency of simulation debriefing. Emerging work suggests that AI technologies may support several debriefing-related processes. Speech recognition and natural language processing may be used to transcribe simulation interactions and analyse communication or teamwork patterns. Generative AI may support facilitators by producing structured feedback, reflective prompts, or summary reports, while conversational AI may guide learners through reflective dialogue. These applications suggest potential for more structured, timely, and data-informed debriefing. However, they also raise concerns regarding accuracy, transparency, bias, data privacy, and the need for facilitator judgement when interpreting learner performance and team interaction [14–17].

Despite growing interest in AI across healthcare simulation and education, the specific role of AI in supporting the debriefing phase of simulation-based healthcare education has not yet been comprehensively synthesised. Existing reviews have examined AI in medical education, healthcare simulation, or simulation-based nursing education more broadly [5, 11, 12, 18, 19], while conceptual work has outlined potential domains of AI application within simulation-based education, including evaluation and feedback processes [13]. However, the literature specifically addressing AI-supported debriefing remains fragmented across disciplines, technologies, and educational contexts. Accordingly, this scoping review aimed to map the existing literature on the use of artificial intelligence to support debriefing in simulation-based healthcare education.

## Methods

This scoping review was conducted in accordance with Arksey and O’Malley’s methodological framework, with subsequent refinements by Levac et al. and guidance from the Joanna Briggs Institute. The review was reported in line with the PRISMA-ScR checklist, provided in Supplementary Material 1.

### Stage 1: identifying the research question

The research question was developed using the Population, Concept, and Context framework. The Population comprised healthcare learners, educators, facilitators, instructors, or teams involved in simulation-based education. The Concept was the use of artificial intelligence to support debriefing-related processes, including feedback generation, simulation performance review, communication or teamwork analytics, reflective dialogue, facilitator support, and structured post-simulation review. The Context was simulation-based healthcare education, including in-person, virtual, immersive, screen-based, or technology-enhanced simulation activities. Guided by this framework, the review addressed the following question:

What is known about the use of artificial intelligence to support debriefing in simulation-based healthcare education?

To operationalise this question, the review explored:

1. the purposes for which artificial intelligence has been applied in debriefing.
2. the types of artificial intelligence methods and tools reported; and
3. the reported outcomes, benefits, limitations, and implementation considerations.

### Stage 2: identifying relevant studies

#### Eligibility criteria

Eligibility criteria were developed using the Population, Concept, and Context framework and are summarised in Table 1. For this review, simulation-based healthcare education was defined as educational activities that use simulated clinical scenarios, environments, patients, or technologies to support healthcare learning and performance development. Debriefing-related processes were defined as activities explicitly connected to post-simulation reflection, feedback, performance review, facilitator-guided discussion, learner self-assessment, or structured analysis of simulation performance. Studies focusing on feedback were included only when the feedback was directly linked to simulation debriefing or post-simulation reflective review. Artificial intelligence was defined as computational systems designed to perform tasks typically requiring human intelligence, including machine learning, natural language processing, generative artificial intelligence, and artificial intelligence-driven analytics. Machine learning referred to systems that identify patterns from data to support prediction, classification, or analysis. Natural language processing referred to computational techniques used to analyse spoken or written language. Generative artificial intelligence referred to systems capable of producing text, feedback, prompts, summaries, or reports. Artificial intelligence-driven analytics referred to automated or semi-automated analysis of learner, team, communication, or performance data.

**Table 1:** Inclusion and exclusion criteria

Framework	Inclusion Criteria	Exclusion Criteria
Population	Studies involving healthcare learners, educators, facilitators, instructors, or teams across undergraduate, postgraduate, continuing professional development, or interprofessional healthcare education.	Studies involving non-healthcare disciplines, non-educational populations, or patient/public education not linked to healthcare professional training.
Concept	Studies reporting the use of artificial intelligence, including machine learning, natural language processing, generative artificial intelligence, or artificial intelligence-driven analytics, to support debriefing-related processes. These included post-simulation feedback, performance review, communication or teamwork analytics, reflective dialogue, facilitator support, structured debriefing reports, or debriefing prompts.	Studies examining artificial intelligence in simulation without explicit reference to debriefing, post-simulation feedback, performance review, or reflective learning; studies describing non-artificial intelligence technologies; studies using artificial intelligence solely for assessment, prediction, or scenario design without a debriefing-related component.
Context	Studies conducted within simulation-based healthcare education, including in-person, virtual, screen-based, immersive, or technology-enhanced simulation activities.	Clinical debriefings not conducted for educational purposes; non-simulation educational contexts; clinical decision-support studies not linked to simulation-based education.
Study Design	Empirical research studies, including qualitative, quantitative, mixed-methods, pilot, feasibility, implementation, evaluation, and educational innovation studies describing an actual artificial intelligence application.	Editorials, commentaries, letters, opinion pieces, narrative reviews, systematic reviews, conference abstracts, theses, dissertations, protocols, and preprints.
Publication Characteristics	English-language, peer-reviewed publications with no date restrictions.	Non-English publications and non-peer-reviewed literature.

#### Search strategy

A comprehensive search strategy was developed and implemented in three phases, with advice from an academic librarian. First, an exploratory search was conducted in Google Scholar, CINAHL, and PROSPERO to identify relevant articles and examine text words in titles and abstracts, as well as index terms used to describe relevant literature. These terms informed the final search strategy. Second, a comprehensive search was conducted in MEDLINE via Ovid, Scopus, Web of Science, and CINAHL. The search combined controlled vocabulary, where available, and free-text keywords related to artificial intelligence, debriefing, feedback, and simulation-based healthcare education. Boolean operators were used to combine search terms and optimise sensitivity and specificity. No date limits were applied because of the emerging nature of artificial intelligence technologies. Searches were limited to English-language publications because of resource constraints. The full MEDLINE search strategy is provided in Supplementary Material 2. Finally, supplementary searches were conducted using selected grey literature sources, guided by the Canadian Agency for Drugs and Technologies in Health Grey Matters tool [20]. The reference lists of included studies were also manually screened to identify additional relevant publications.

### Stage 3: study selection

All records retrieved from the searches were exported to reference management software, and duplicates were removed before screening. Titles and abstracts of the remaining records were screened against the inclusion criteria by the first reviewer. Where relevance was unclear, full texts were retrieved and discussed with the second reviewer to support consistency in eligibility decisions. Full-text articles were assessed against the inclusion criteria, and any uncertainties were resolved through discussion and consensus. Studies that met the inclusion criteria were included in the data-charting stage.

### Stage 4: data charting

A structured data-charting form was developed to extract information relevant to the review question and objectives. The form captured descriptive study characteristics, including author, year, country, study design, participants, simulation context, artificial intelligence method or tool, purpose of artificial intelligence use in relation to debriefing, reported outcomes, and limitations or implementation considerations. Data were charted by the first reviewer and checked by the second reviewer for consistency and completeness. Any uncertainties were discussed and resolved by consensus.

### Stage 5: collating, summarising, and reporting the results

The findings were synthesised descriptively and thematically. A descriptive numerical summary was used to report study characteristics, including publication year, country, study design, participants, simulation context, and type of artificial intelligence application. Thematic synthesis was then used to group studies according to how artificial intelligence supported debriefing-related processes. Findings were organised around the purposes of artificial intelligence use, types of artificial intelligence methods and tools, reported outcomes, and implementation or ethical considerations.

### Reflexivity

As reviewers exploring literature at the intersection of artificial intelligence, simulation-based education, and debriefing, we acknowledge that our academic backgrounds and experience in healthcare education and simulation may have shaped how we interpreted and synthesised the available evidence. Reflexivity recognises that researchers’ perspectives and prior knowledge can influence the interpretation of scholarly work and should therefore be acknowledged to enhance transparency in the review process [21, 22]. To support transparency and consistency, the review followed established scoping review methodological frameworks and reporting guidance [23–27]. Eligibility uncertainties were discussed between reviewers, and data charting was checked for consistency and completeness. Reflexive discussion was used during synthesis to consider how studies were categorised and interpreted.

## Results

**Fig. 1:**
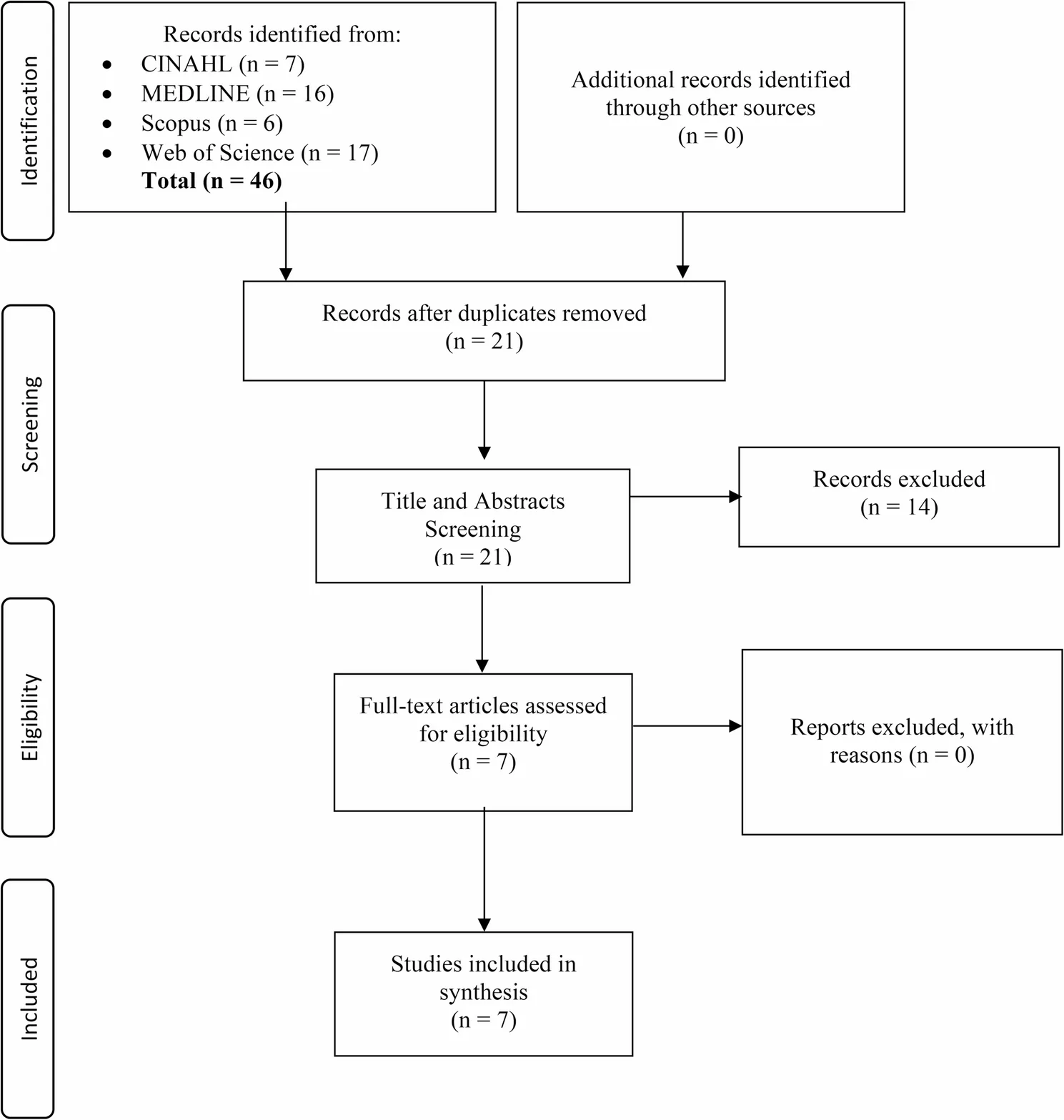
PRISMA-ScR flow diagram

A total of 46 records were identified through database searches. No additional records were identified through supplementary searches or reference-list screening. After removal of 25 duplicates, 21 records remained for title and abstract screening. Fourteen records were excluded based on the eligibility criteria. The full texts of seven articles were assessed for eligibility, and all met the inclusion criteria. Consequently, seven studies were included in the review. The study selection process is illustrated in the PRISMA-ScR flow diagram (Fig. 1).

### Study characteristics

Seven studies published between 2023 and 2026 met the inclusion criteria [28–34]. The studies were conducted in the United States (*n* = 3), Switzerland (*n* = 2), Chile (*n* = 1), and South Korea (*n* = 1). Study designs were heterogeneous and included pilot or feasibility studies, cross-sectional research, qualitative exploratory research, model development and validation, mixed-methods evaluation, and a within-subjects pre–post simulation study. A summary of the included studies is presented in Table 2. All included studies were conducted in simulation-based healthcare education contexts. Simulation formats included in-person team-based simulation, remote interprofessional simulation, screen-based virtual simulation, immersive medical simulation, and structured nursing simulation debriefing. Participants included undergraduate nursing students, medical students, resident and attending physicians, nurses, interprofessional trauma or perioperative teams, simulation instructors, and simulation facilitators.

**Table 2:** Characteristics of included studies

Author, year, country	Study design and participants	Simulation/debriefing context	AI method/tool	Role of AI in debriefing	Key outcomes and limitations
Brutschi et al., 2024, Switzerland [28]	Observational feasibility study; third-year medical students in team-based simulation, with two experienced debriefers.	Post-simulation team debriefings following patient handover simulation.	Machine learning-based speaker diarisation and sociogram generation.	Analysed speakers, speech duration, and interaction patterns during debriefing.	Demonstrated technical feasibility and high speaker-diarisation accuracy; limited by a small number of debriefings and the same instructors across sessions.
Vaughn et al., 2025, United States [31]	Pilot feasibility and usability study; six nursing simulation facilitators.	Facilitator debriefing practice in nursing simulation.	Generative artificial intelligence chatbot using retrieval-augmented generation.	Provided evidence-informed, personalised feedback on facilitator debriefing skills.	Feasible and perceived as useful and easy to use; limited by small convenience sample, single institution, and early-stage prototype testing.
Jang et al., 2025, Republic of Korea [33]	Model development and validation study with convergent mixed-method evaluation; fourth-year nursing students, instructors, and experts.	Nursing simulation debriefing using question-centred learning.	ChatGPT 4.0 and structured prompt engineering.	Supported question-centred debriefing prompts and clinical reasoning.	Reported improvements in artificial intelligence competency, class interest, knowledge, and confidence; limited by small sample, single university, and one-group design.
Gonzalez and Nagendran, 2025, United States [34]	Pilot mixed-methods study; 52 undergraduate nursing students.	Screen-based virtual nursing simulation followed by artificial intelligence-facilitated debriefing.	Conversational artificial intelligence and voice-based analysis.	Guided asynchronous learner reflection using structured debriefing dimensions.	No significant correlation between time in debriefing and grades; suggested potential for scalable reflective practice but further validation is needed.
Rosser et al., 2023, United States [29]	Within-subjects pre–post simulation study; 30 interprofessional trauma teams involving 150 participants.	Trauma simulations conducted before and after debriefing.	Natural language processing-based discourse coding algorithms.	Analysed changes in teamwork-related communication during subsequent post-debrief simulation performance.	Identified post-debrief improvements in several automatically coded communication behaviours; limited by manual transcription and simplified coding algorithms.
Armijo-Rivera et al., 2026, Chile [30]	Cross-sectional relational analytic study; 15 novice healthcare simulation instructors.	Remote interprofessional simulation debriefings.	GailBot API for automated transcription and artificial intelligence-based speech metrics.	Quantified instructor speaking time and facilitation patterns during debriefing.	Instructors spoke for an average of 56.09% of debriefing time; higher instructor talk time correlated negatively with DASH self-ratings; limited by small sample and cross-sectional design.
Tscholl et al., 2026, Switzerland [32]	Qualitative exploratory study; 41 healthcare professionals, 26 artificial intelligence-generated reports, and 27 learner interviews.	Immersive medical simulation with facilitator-led debriefing.	Artificial intelligence-assisted speech recognition, Isaac, and ChatGPT-4o using the Team-FIRST framework.	Generated structured teamwork reports to support facilitator-led debriefing.	Experts valued transcripts, illustrative quotes, and structured feedback; limitations included speaker misattribution, overgeneralisation, lack of non-verbal/contextual data, and need for human facilitation.

*AI* artificial intelligence, *DASH* Debriefing Assessment for Simulation in Healthcare, *LLM* large language model, *NLP* natural language processing, *RAG* retrieval-augmented generation

### AI applications in debriefing-related processes

Artificial intelligence applications were grouped into three broad categories according to their function within debriefing-related processes: communication and performance analytics, facilitator or instructor support, and learner-facing reflective dialogue. Three studies used speech recognition, speaker diarisation, or natural language processing to analyse communication and interaction patterns during simulation or debriefing [28–30]. Speaker diarisation refers to the automated process of identifying and separating different speakers in an audio recording. Brutschi et al. used speaker diarisation to analyse debriefing interaction patterns and generate sociogram visualisations [28]. Armijo-Rivera et al. used the GailBot API to quantify novice instructors’ speaking time and facilitation patterns during debriefing [30]. Rosser et al. used natural language processing to analyse trauma team discourse during simulations conducted before and after debriefing, thereby examining changes in teamwork-related communication behaviours during subsequent simulation performance [29].

Three studies used generative artificial intelligence to support facilitator or instructor activities [31–33]. Vaughn et al. developed a generative artificial intelligence chatbot with retrieval-augmented generation to provide evidence-informed feedback on facilitator debriefing skills [31]. Tscholl et al. used artificial intelligence-assisted speech recognition and large language model-based analysis to generate structured teamwork reports designed to support facilitator-led debriefing [32]. Jang et al. developed and validated a question-centred prompt engineering model to support nursing simulation debriefing [33]. Two studies included learner-facing reflective components [33, 34]. Gonzalez and Nagendran explored artificial intelligence-facilitated debriefing following a screen-based nursing simulation, with learners engaging in structured reflective dialogue [34]. Jang et al. integrated generative artificial intelligence and prompt engineering into a structured nursing simulation debriefing model to support question-centred learning and clinical reasoning [33]. Across the included studies, artificial intelligence was most commonly positioned as an adjunct to human facilitation. Adjunct uses included facilitator feedback, teamwork report generation, communication analytics, instructor speech analysis, and structured debriefing prompts [28–33]. More autonomous learner-facing uses were less common and were reported mainly in pilot or developmental contexts [33, 34].

### Reported outcomes, benefits, limitations, and implementation considerations

Reported outcomes were primarily feasibility-, usability-, perception-, and early educational outcome measures. Brutschi et al. reported high accuracy in speaker diarisation and demonstrated the feasibility of generating visual interaction patterns from debriefing audio [28]. Rosser et al. reported post-debrief improvements in automatically coded teamwork communication behaviours, including verbalising findings, acknowledging communication, directed communication, directing assessment and role assignment, and leader-as-hub communication [29]. Armijo-Rivera et al. found that novice instructors spoke for an average of 56.09% of debriefing time and that higher instructor talk time was negatively correlated with DASH self-ratings [30]. Generative artificial intelligence studies reported feasibility and perceived usefulness in supporting debriefing-related tasks. Vaughn et al. reported that a generative artificial intelligence chatbot designed to provide feedback on facilitator debriefing skills was feasible and perceived as useful and easy to use [31]. Tscholl et al. found that simulation experts valued artificial intelligence-generated reports for providing detailed transcripts, illustrative quotes, and structured feedback to support debriefing. However, limitations included speaker misattribution, inaccurate categorisation, overgeneralised interpretations, and absence of contextual or non-verbal data [32]. Jang et al. reported that a question-centred prompt engineering model demonstrated content validity and was associated with improvements in artificial intelligence competency, class interest, knowledge, and confidence among nursing students [33].

Learner-facing artificial intelligence debriefing studies reported mixed findings. Gonzalez and Nagendran found no significant correlation between time spent in artificial intelligence-facilitated debriefing and student grades, although students who met more reflective dimensions spent less time in debriefing than those who met fewer dimensions [34]. Jang et al. reported improvements in learner outcomes following implementation of a question-centred prompt engineering model, but the study was limited by a small sample from one university and a single-group design [33]. Across the included studies, common limitations included small sample sizes, single-institution settings, pilot or exploratory designs, limited comparison groups, and lack of long-term outcome evaluation [30–34]. Implementation considerations included transcription accuracy, audio quality, speaker identification, data privacy, transparency of artificial intelligence-generated outputs, algorithmic bias, and the need for human interpretation of artificial intelligence-generated feedback or analytics [28, 30, 32, 34]. Overall, the included studies positioned artificial intelligence primarily as an adjunct to human facilitation rather than as a replacement for the facilitator role [28, 30–34].

## Discussion

This scoping review mapped the emerging literature on the use of artificial intelligence to support debriefing in simulation-based healthcare education. Across the seven included studies, AI was used in three main ways: to analyse communication and performance data, to support facilitators or instructors through structured feedback and report generation, and to guide learner-facing reflective dialogue. Overall, the evidence suggests that AI is currently positioned mainly as an adjunct to human facilitation rather than as a replacement for the facilitator role [28–34].

A key contribution of AI-supported debriefing lies in its potential to support performance-focused debriefing. Traditional debriefing depends heavily on facilitator observation, memory, and interpretation, which may be challenged by complex team dynamics, high cognitive load, and the volume of performance data generated during simulation. AI-supported analytics may help facilitators identify patterns that are difficult to capture through observation alone, such as participation distribution, speaking time, communication flow, role clarity, and changes in teamwork behaviours [28–30]. This is consistent with wider developments in data-informed debriefing, where objective performance data, such as CPR quality metrics, have been used to guide debriefing and improve selected performance outcomes in simulated cardiac arrest. For example, Cheng et al. reported that data-informed debriefing improved several CPR quality metrics compared with traditional debriefing in simulated paediatric cardiac arrest, although it did not improve time to critical interventions [35]. This suggests that AI may extend performance-focused debriefing by enabling more detailed, timely, and scalable analysis of both technical and non-technical performance indicators. The included studies also indicate that generative AI may support facilitator preparation and faculty development. Tools such as generative AI chatbots, structured feedback interfaces, and AI-generated teamwork reports may help facilitators organise observations, generate reflective prompts, and identify issues for discussion [31–33]. Such tools may be especially useful for novice facilitators or settings where access to expert debriefing coaching is limited. However, AI-generated outputs require careful interpretation. The included studies reported limitations such as speaker misattribution, inaccurate categorisation, overgeneralised interpretations, and absence of contextual or non-verbal data [28, 30, 32]. These limitations are important because effective debriefing requires more than the identification of observable behaviours; it also requires facilitator judgement, psychological safety, contextual interpretation, and sensitivity to learners’ emotional and cognitive needs.

Learner-facing AI applications may also support reflective practice, particularly in asynchronous or virtual simulation contexts. AI-facilitated dialogue and prompt-engineered debriefing models may provide structured opportunities for learners to revisit simulation experiences, respond to reflective prompts, and consider their clinical reasoning [33, 34]. However, current evidence remains preliminary. Most studies focused on feasibility, usability, technical accuracy, perceived usefulness, or short-term educational outcomes, with limited evidence of sustained learning, behavioural transfer, or clinical impact. Therefore, AI-facilitated reflection should be viewed as a complementary approach rather than a substitute for skilled human-led debriefing.

Ethical and implementation issues remain central to the responsible use of AI in simulation debriefing. AI-supported debriefing may involve audio recordings, transcripts, learner performance data, and automated interpretation of team behaviours. This raises questions about consent, data privacy, transparency, algorithmic bias, and how AI-generated outputs should be communicated to learners. These concerns are particularly important in debriefing, where psychological safety and trust are essential. Institutions implementing AI-supported debriefing should therefore ensure clear governance processes, human-in-the-loop review, faculty development, and transparent communication with learners about how AI tools are used. This review highlights the need for more rigorous research. Future studies should move beyond single-institution pilot work and evaluate AI-supported debriefing using comparative, multi-site, and longitudinal designs. Research should examine not only feasibility and user perceptions, but also objective outcomes such as debriefing quality, learner reflection, communication behaviours, clinical reasoning, teamwork performance, and transfer to practice. Further work is also needed to clarify which aspects of debriefing are appropriate for AI support and which should remain firmly within the domain of human facilitation.

### Strengths and limitations

This scoping review was conducted in accordance with established methodological guidance and reported in line with the PRISMA-ScR checklist. The review used a structured search across multiple databases, supplementary searching, and a predefined Population, Concept, and Context framework to guide eligibility decisions. The review also provides a focused synthesis of an emerging area of simulation-based healthcare education by mapping how artificial intelligence has been applied specifically to support debriefing-related processes. Several limitations should be acknowledged. First, initial title and abstract screening was conducted by one reviewer, with consultation from a second reviewer when eligibility decisions were unclear. Although this approach was supported by discussion and consensus, dual independent screening may have reduced the risk of missing relevant studies or introducing selection bias. Second, the search was limited to English-language publications, which may have excluded relevant studies published in other languages. Third, although supplementary searches were conducted, unpublished or ongoing work may not have been fully captured. In addition, formal critical appraisal of included studies was not undertaken, which is consistent with scoping review methodology but limits conclusions about the quality of the evidence. The included studies were also heterogeneous in design, setting, participant groups, artificial intelligence tools, and reported outcomes. Most were small-scale, exploratory, or single-institution studies, limiting the generalisability of the findings. Therefore, the review should be interpreted as a descriptive mapping of the current evidence rather than a definitive evaluation of the effectiveness of artificial intelligence-supported debriefing.

### Implications and recommendations

The findings suggest that artificial intelligence may support debriefing by providing additional data for performance review, generating structured feedback or reports, and supporting reflective dialogue. However, current evidence remains preliminary, and implementation should be guided by pedagogical purpose rather than technological novelty. AI-supported debriefing should therefore be used as an adjunct to skilled facilitation, with educators retaining responsibility for interpreting AI-generated outputs within the clinical, educational, and emotional context of the simulation. For practice, institutions considering AI-supported debriefing should establish clear governance processes addressing consent, data privacy, transparency, learner trust, and human review of AI-generated feedback. Faculty development is also needed to support educators in interpreting AI-derived analytics and integrating them appropriately into debriefing conversations. For research, future studies should move beyond feasibility and perception-based outcomes to evaluate the effect of AI-supported debriefing on debriefing quality, learner reflection, clinical reasoning, teamwork behaviours, and transfer to practice. Multi-site, comparative, and longitudinal studies are needed to determine when and how AI adds value to simulation-based healthcare education.

## Conclusion

This scoping review mapped the emerging literature on the use of artificial intelligence to support debriefing in simulation-based healthcare education. The findings indicate that artificial intelligence is currently being applied to support communication and performance analytics, facilitator or instructor support, and learner-facing reflective dialogue, but the evidence remains limited, exploratory, and largely single-institutional. Further rigorous research is needed to evaluate the educational effectiveness, ethical implementation, and appropriate integration of artificial intelligence as an adjunct to human facilitation in simulation debriefing.

## Supplementary Information

Supplementary Material 1.

Supplementary Material 2.

## Data Availability

No new datasets were generated. All data charted and synthesised in this review are derived from published studies and are included in this article and its supplementary materials.

## Abbreviations

AI: Artificial Intelligence
CADTH: Canadian Agency for Drugs and Technologies in Health
CINAHL: Cumulative Index to Nursing and Allied Health Literature
MEDLINE: Medical Literature Analysis and Retrieval System Online
MeSH: Medical Subject Headings
ML: Machine Learning
NLP: Natural Language Processing
Ovid: Ovid Technologies database platform
PCC: Population, Concept, Context
PRISMA-ScR: Preferred Reporting Items for Systematic Reviews and Meta-Analyses Extension for Scoping Reviews
VR: Virtual Reality
