# Supplementary material for: Artificial intelligence to support debriefing in simulation-based healthcare education: a scoping review
Source: Adv Simul (Lond). 2026 Jun 25;11:60. doi: 10.1186/s41077-026-00447-6 (PMC13560297; doi:10.1186/s41077-026-00447-6)
Supplement: Supplementary file 1 [file 41077_2026_447_MOESM2_ESM.pdf]

exp Artificial Intelligence/
exp Machine Learning/
exp Natural Language Processing/
exp Deep Learning/
(artificial intelligence or AI).ti,ab,kf.
(machine learning or deep learning).ti,ab,kf.
(natural language processing or NLP).ti,ab,kf.
(generative AI or generative artificial intelligence).ti,ab,kf.
(large language model* or LLM or LLMs).ti,ab,kf.
(ChatGPT or GPT-3 or GPT-4 or transformer model*).ti,ab,kf.
(AI-driven or AI-based or AI-assisted).ti,ab,kf.
or/1-11
Debrief*.mp.
(debrief* or "after action review*" or AAR).ti,ab,kf.
(post-simulation discussion or reflective discussion*).ti,ab,kf.
(structured reflection or facilitated reflection).ti,ab,kf.
or/13-16
exp Simulation Training/
exp Patient Simulation/
exp Manikins/
(simulation-based or simulation training or simulated scenario*).ti,ab,kf.
(high-fidelity simulation or low-fidelity simulation).ti,ab,kf.
(clinical simulation or healthcare simulation).ti,ab,kf.
or/18-23
exp Education, Medical/
exp Education, Nursing/
exp Students, Health Occupations/
exp Clinical Competence/
(healthcare education or medical education or nursing education or health professions education).ti,ab,kf.
(clinical training or professional development).ti,ab,kf.
exp Education, Public Health Professional/
or/25-31
12 and 17 and 24 and 32
